# Catgut Versus Polypropylene Sutures for Transcolumellar Incision Closure in Open Rhinoplasty: A Retrospective Cohort Study

**DOI:** 10.7759/cureus.9769

**Published:** 2020-08-15

**Authors:** Saud Aldhabaan, Jibril Y Hudise, Mohammed ALqarny, Ahmed Alarfaj

**Affiliations:** 1 Otolaryngology, King Faisal Medical City, Abha, SAU; 2 Otolaryngology, King Abdulaziz University Hospital, Riyadh, SAU; 3 Otolaryngology, University of Bisah, Bisha, SAU

**Keywords:** rhinoplasty, transcolumellar incision, catgut suture, polypropylene suture

## Abstract

Background

Open rhinoplasty, including the transcolumellar approach, is commonly performed as it has fewer side effects and provides superior anatomical control to the surgeons compared to closed rhinoplasty. However, the postoperative scar outcomes, such as scar appearance, vary depending on the type of suture used in wound closure, and the optimal suture type is not firmly established.

Objective

To compare the impact of catgut versus polypropylene sutures on the postoperative transcolumellar scar outcomes and patient satisfaction following open rhinoplasty.

Methods

This retrospective cohort study, including 100 patients who underwent transcolumellar open rhinoplasty, was conducted at otolaryngology department of King Abdulaziz University Hospital, Riyadh, KSA. The patients were divided into two groups: the propylene suture group (group 1), which included 15 males and 35 females with a mean age of 31.5 years and underwent surgery using propylene sutures, and the catgut suture group (group 2), which included 10 males and 40 females with a mean age of 30.5 years and underwent surgery using catgut sutures. The postoperative transcolumellar scar outcomes, as determined by visual analogue scale (VAS) and Stony Brook Scar Evaluation Scale (SBSES) scores, and patient satisfaction, as assessed using a self-assessment scale, were compared between groups.

Results

The scars were unnoticeable in the majority of both groups: 88% in group 1 and 86% in group 2. The VAS and SBSES scores did not significantly differ between groups. Patients' satisfaction rates were also comparable and did not significantly differ between groups (p = 0.341).

Conclusion

Both catgut and polypropylene sutures lead to similar outcomes and patient satisfaction rates in terms of postoperative rhinoplasty transcolumellar scars. Thus, catgut may be the optimal suture for closing transcolumellar incisions following open rhinoplasty.

## Introduction

Rhinoplasty is a common surgical intervention aiming to preserve the aesthetic and functional properties of the nose. This surgery is also applied to reconstruct the deformed nose resulting from traumatic injuries, maxillary surgeries, or birth defects [1-3]. There are two main approaches to rhinoplasty: open and closed rhinoplasty. Open rhinoplasty is performed externally via an alar base or transcolumellar incision [4]. The transcolumellar scar provides superior anatomical and surgical control and generally heals well without complications using non-absorbable monofilament sutures, such as polypropylene [5]. However, follow-up visits are required to remove these sutures, leading to significant discomfort [6]. Absorbable sutures that can resorb spontaneously without affecting the aesthetics of the scar are a viable alternative [7,8]. Catgut is an absorbable, monofilament suture that consists of organic material derived from animal intestines, and can therefore elicit an allergic reaction and affect wound healing [9].

However, Sharma and Mehrotra reported that surgeons' fear of using catgut is unwarranted as it led to complications such as wound dehiscence and infection in only 15 (4.3%) of 350 surgeries and provided excellent scar healing in 322 (92%) cases [10]. In contrast, polypropylene is a synthetic suture that causes a limited allergic reaction and does not adhere to the tissues. However, it can result in wound sinus formation, pain, palpable knots, and rate of wound infection can be up to 24% [11]. Previous studies investigating the use of absorbable and non-absorbable sutures in open rhinoplasty reported comparable results with both sutures. Thus, the optimal suture type has not been established [6-8].

An ideal suture is cheap, reliable, does not cut through the skin, provides superior handling and anatomical control during surgery, and promotes excellent wound healing post-surgery [12]. In the present study, we aimed to compare the impact of catgut and polypropylene sutures on postoperative scar outcomes and patients' satisfaction.

## Materials and methods

Patients

This retrospective cohort study was done to assess the postoperative wound scar outcomes and satisfaction rates with the use of catgut versus polypropylene sutures in patients who underwent tanscolumellar open rhinoplasty. We retrospectively looked into patient procedure records of open rhinoplasty using transcolumellar approach done by two surgeons and performed postoperative photography on the scars of the patients. The study was conducted at otolaryngology department of King Abdulaziz University Hospital, Riyadh, KSA between May 2019 and May 2020. 

The study included patients who were 18 years or above and underwent open rhinoplasty for improving nose aesthetics with transcolumellar incision and the wound closure was done using catgut or polypropylene sutures. The exclusion criteria include patients below 18 years, patients who spent less than one year following their surgery, or had a rhinoplasty done secondary to trauma or congenital anomalies.

Assessment

The cases were followed-up after a year via telephone consultation, and self-assessment tools were used to assess the healing and satisfaction rates among the patients. A visual analogue scale (VAS) was used to categorize the postoperative scar outcome into unnoticeable, noticeable but acceptable, and noticeable and not acceptable. Assessment of patient photography (basal view) after one year of surgery, by two rhinoplastic surgeons (the same surgeons who did the surgeries) using Stony Brook Scar Evaluation Scale (SBSES) was carried out in order to categorize scar outcomes as poor (score 0-1), moderate (score 2-3), and good (score 4-5) [13]. Patient satisfaction was measured using a self-assessment scale ranging from 0 to 10, where 0 indicated complete dissatisfaction and 10 indicated total satisfaction. Any disagreement among the assessors was resolved through mutual consultation and concurrence.

Statistical analysis

Statistical Package for the Social Sciences (SPSS), v 21.0 (IBM Corp., Armonk, NY) was used to analyze the data. The Chi-squared test was used to evaluate statistical significance, and a p-value of ≤0.05 was considered significant. 

## Results

Our study included 100 participants who were divided into two groups. Group 1 included 50 participants who underwent transcolumellar incision surgery using interrupted polypropylene sutures. This group had a mean age of 31.4±6.5 years and included 35 (70%) females and 15 (30%) males. Group 2 underwent transcolumellar incision surgery using catgut sutures. This group had a mean age of 30.5±6.3 years and included 40 (80%) females and 10 (20%) males (Table 1).

**Table 1:** Patient demographics

Variables	Group 1 (Polypropylene)	Group 2 (Catgut)
Mean age (years)	31.4±6.5	30.5±6.3
Sex		
Females, n (%)	35 (70%)	40 (80%)
Males, n (%)	15 (30%)	10 (20%)

. 

We used the VAS to assess the patients’ perception of their scars. In group 1, 44 (88%), five (10%), and one (2%) patients reported having unnoticeable, noticeable but acceptable, and noticeable and unacceptable scars, respectively. In group 2, 43 (86%) and seven (14%) patients reported having unnoticeable and noticeable but acceptable scars, respectively. None of the participants in group 2 reported having noticeable and unacceptable scars. The differences between groups regarding patients’ perception of the postoperative scar outcome were not significant (p = 1.000) (Table 2). 

**Table 2:** VAS and SBSES analysis for both groups VAS, visual analogue scale; SBSES, Stony Brook Scar Evaluation Scale

Visual Analogue Scale (VAS) analysis				
Group	Unnoticeable scars, n (%)	Noticeable but acceptable, n (%)	Noticeable and unacceptable, n (%)	p-value
Group 1	44 (88%)	5 (10%)	1 (2%)	1.000
Group 2	43 (86%)	7 (14%)	0 (0)	
Stony Brook Scar Evaluation Scale (SBSES) analysis				
	Poor score (0-1), n (%)	Moderate score (2-3), n (%)	Good score (4-5), n (%)	p-value
Group 1	1 (2%)	6 (12%)	43 (86%)	0.221
Group 2	0 (0%)	5 (10%)	45 (90%)	

Table 2 summarizes the SBSES scores. The majority of patients in both groups rated their scars outcomes as ‘good’ (scored 4 or 5) (p = 0.225).

When asked to rate their satisfaction regarding scar outcomes on a scale of 0-10, most of the participants in both groups reported satisfaction scores of ≥8. The mean satisfaction scores were 9.0 and 9.2 for groups 1 and 2, respectively (p = 0.341) (Table 3).

**Table 3:** Satisfaction scores from both groups

Group	Lowest Rating	Highest Rating	Mean rating	Standard deviation	p-value
Group 1	7.0	10.0	9.0800	0.82906	0.341
Group 2	7.0	10.0	9.2200	0.84007	

## Discussion

The main purpose of the current study was to determine the impact of two different suture materials on aesthetic outcomes and patient satisfaction following open rhinoplasty. Our study showed that both suture types did not differ in terms of their aesthetic outcomes and patient satisfaction.

Ashraf et al. studied scar healing and patient satisfaction following transcolumellar septorhinoplasty. The sutures used in the study were non-absorbable Prolene® and absorbable Vicryl Rapide®. The study did not report any statistically significant (p=0.39) difference in scar rating and patient satisfaction with the use of either suture materials [14]. In another report, Alijanpour et al. analyzed 15 research papers comparing absorbable and non-absorbable sutures for outcomes including wound aesthetics and comfort in patients undergoing open rhinoplasty. The authors concluded that both suture types produced similar scar aesthetics. However, the patients reported greater satisfaction with the use of absorbable sutures because these sutures did not require removal [15]. This aspect has been discussed in several other studies wherein researchers did not notice any difference between aesthetic outcomes with the use of absorbable or non-absorbable sutures in ear, nose, and throat or other facial surgeries [16-18]. Our current findings are consistent with the literature as we demonstrated that absorbable and non-absorbable sutures do not differ in terms of wound aesthetics and patient satisfaction scores.

To the best of our knowledge, our study is the first of its kind to use catgut as an absorbable suture in comparison to a non-absorbable suture (polypropylene) for assessing wound healing and patient satisfaction following transcolumellar open rhinoplasty. Surgeons are often concerned about the use of catgut due to the possibility of cross-sensitivity as it contains organic material, which might affect wound healing and aesthetics. However, this concern has not been clinically validated. Gazivado et al. compared wound healing and the incidence of complications such as wound infection and local reactions with the use of different types of absorbable sutures (catgut, Dexon, and Vicryl). The results suggested that none of the suture materials hindered wound healing, and there was a low incidence of wound complications with all of the used suture materials that did not reach statistical significance [19]. Other reports advocate the use of catgut as an absorbable suture as it is absorbable, has good tensile strength, is more comfortable for the patients as it spares them the pain of suture removal, leads to excellent wound healing, and has comparable rates of complications to other absorbable and non-absorbable suture materials [14,15,19,20].

## Conclusions

Our study has demonstrated that both absorbable catgut and non-absorbable polypropylene sutures can be used for the closure of transcolumellar incisions following open rhinoplasty. We found no significant difference in wound healing and patient satisfaction with the use of either of the suture materials. Therefore, catgut may be the optimal suture for closing transcolumellar incisions following open rhinoplasty as it gives comparable aesthetic outcomes to polypropylene and saves surgeons’ time. 
